# Structures of active-state orexin receptor 2 rationalize peptide and small-molecule agonist recognition and receptor activation

**DOI:** 10.1038/s41467-021-21087-6

**Published:** 2021-02-05

**Authors:** Chuan Hong, Noel J. Byrne, Beata Zamlynny, Srivanya Tummala, Li Xiao, Jennifer M. Shipman, Andrea T. Partridge, Christina Minnick, Michael J. Breslin, Michael T. Rudd, Shawn J. Stachel, Vanessa L. Rada, Jeffrey C. Kern, Kira A. Armacost, Scott A. Hollingsworth, Julie A. O’Brien, Dawn L. Hall, Terrence P. McDonald, Corey Strickland, Alexei Brooun, Stephen M. Soisson, Kaspar Hollenstein

**Affiliations:** 1 Computational & Structural Chemistry, MRL, Merck & Co., Inc, Kenilworth, NJ USA; 2 Computational & Structural Chemistry, MRL, Merck & Co., Inc, West Point, PA USA; 3 Screening & Compound Profiling, MRL, Merck & Co., Inc, Kenilworth, NJ USA; 4 Quantitative Bioscience, MRL, Merck & Co., Inc, West Point, PA USA; 5 Discovery Chemistry, MRL, Merck & Co., Inc, West Point, PA USA; 6 Computational & Structural Chemistry, MRL, Merck & Co., Inc., South San Francisco, CA USA; 7 Neuroscience, MRL, Merck & Co., Inc, West Point, PA USA

**Keywords:** G protein-coupled receptors, Orexin, Cryoelectron microscopy

## Abstract

Narcolepsy type 1 (NT1) is a chronic neurological disorder that impairs the brain’s ability to control sleep-wake cycles. Current therapies are limited to the management of symptoms with modest effectiveness and substantial adverse effects. Agonists of the orexin receptor 2 (OX_2_R) have shown promise as novel therapeutics that directly target the pathophysiology of the disease. However, identification of drug-like OX_2_R agonists has proven difficult. Here we report cryo-electron microscopy structures of active-state OX_2_R bound to an endogenous peptide agonist and a small-molecule agonist. The extended carboxy-terminal segment of the peptide reaches into the core of OX_2_R to stabilize an active conformation, while the small-molecule agonist binds deep inside the orthosteric pocket, making similar key interactions. Comparison with antagonist-bound OX_2_R suggests a molecular mechanism that rationalizes both receptor activation and inhibition. Our results enable structure-based discovery of therapeutic orexin agonists for the treatment of NT1 and other hypersomnia disorders.

## Abstract (web summary)

Agonists of the orexin receptor 2 (OX_2_R) show promise in the treatment of narcolepsy. Cryo-EM structures of active-state OX_2_R bound to an endogenous peptide agonist and a small-molecule agonist suggest a molecular mechanism that rationalizes both receptor activation and inhibition.

## Introduction

Orexin A (OxA) and orexin B (OxB), also termed hypocretin-1 and hypocretin-2, respectively, are excitatory neuropeptides produced in the hypothalamus that control sleep/wake behavior by promoting and maintaining wakefulness, and suppressing rapid eye movement (REM) sleep^1–3^. Loss of orexin-producing neurons is the cause of narcolepsy type 1 (NT1), also termed classical narcolepsy or narcolepsy with cataplexy^4,5^, a lifelong neurological disorder that affects 1 in 2000 to 1 in 4000 people^6–8^. Patients suffer from excessive daytime sleepiness and abnormal REM sleep-related symptoms, such as hallucinations, sleep paralysis, and cataplexy. Available therapies are limited to managing symptoms with modest effectiveness and moderate to severe adverse effects^9^.

OxA and OxB signal through the two closely related receptors, orexin receptor type 1 and type 2 (OX_1_R and OX_2_R, respectively), expressed widely across the central nervous system^3^. OX_1_R and OX_2_R are class A G-protein-coupled receptors (GPCRs) of the β-branch that signal predominantly through heterotrimeric G_q/11_ leading to increased cytosolic Ca^2+^ levels^10^. Inhibition of both OX_1_R and OX_2_R with antagonists provides an effective treatment of insomnia with two drugs recently reaching the market^11–14^. Conversely, intracerebroventricular administration of OxA promotes wakefulness and suppresses REM sleep in mice^15^ and, more recently, the discovery of non-peptide orexin agonists have demonstrated potential for the treatment of NT1 and other hypersomnia disorders^16–18^, by targeting the orexin system via selective activation of OX_2_R. Despite progress with two OX_2_R-selective agonists in early clinical trials^19^, identification of efficacious oral small-molecule agonists with drug-like properties remains challenging.

**Fig. 1:**
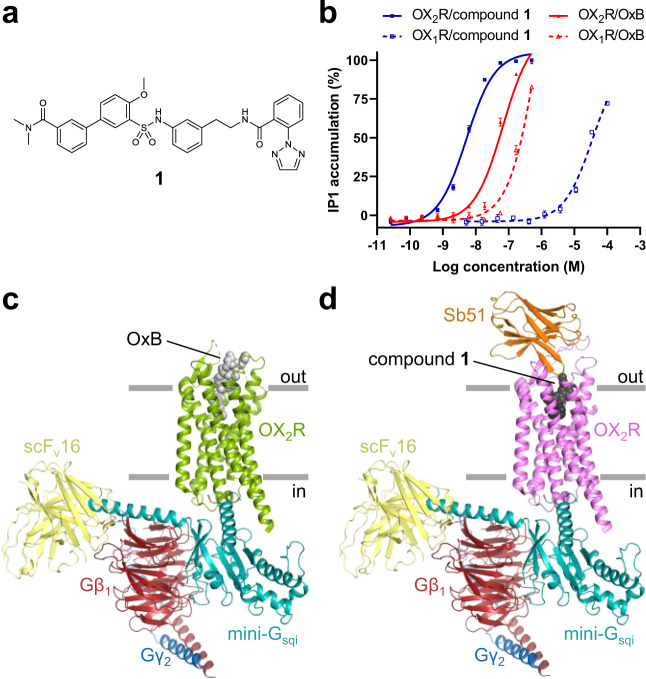
Overall structures of peptide agonist and small-molecule agonist-activated OX_2_R. **a** Structural formula of the small-molecule orexin agonist used in this study. **b** Concentration-dependent activation of OX_1_R and OX_2_R by OxB and compound **1**. Half maximal effective concentrations represented as pEC_50_ are: 4.45 ± 0.06 (OX_1_R–compound **1**), 8.28 ± 0.03 (OX_2_R–compound **1**), 6.09 ± 0.14 (OX_1_R–OxB), and 7.17 ± 0.07 (OX_2_R–OxB); error bars represent the standard error of the mean of *n* = 4 independent experiments for all except for OX_1_R/compound **1**, for which *n* = 5 independent experiments were performed. Data are presented as mean values ± s.e.m. **c**, **d** Structures of nucleotide-free OX_2_R–G-protein complexes bound to OxB and compound **1**, respectively, color-coded by subunit. Source data are provided as a Source Data file.

Recently reported structures of antagonist-bound OX_1_R and OX_2_R^20–23^ have greatly advanced our understanding of the molecular basis of antagonist recognition and subtype selectivity. Yet, their utility for dissecting the molecular mechanism of activation and for driving the discovery of small-molecule agonists has remained limited. In this work, we determined single-particle cryo-electron microscopy (cryo-EM) structures of OX_2_R–G-protein complexes bound to OxB and the small-molecule agonist 3′-(*N*-(3-(2-(2-(2H-1,2,3-triazol-2-yl)benzamido)ethyl)phenyl)sulfamoyl)-4′-methoxy-*N*,*N*-dimethyl-[1,1′-biphenyl]-3-carboxamide (compound **1**; Fig. 1a) to further our understanding of orexin signaling and to enable structure-based drug discovery of novel therapeutics for the treatment of NT1. Our results shed light on the molecular details of peptide and small-molecule agonist recognition, reveal the global and local conformational changes associated with receptor activation, and suggest a molecular mechanism for activation by peptide and small-molecule agonists.

## Results

### Structure determination

We reconstituted an OX_2_R–G-protein complex from purified subunits, using an engineered variant of human OX_2_R with a truncated carboxy-terminus and a shortened intracellular loop (ICL) 3. This construct enabled high-level heterologous expression and purification of homogeneous material, while maintaining wild-type-like affinity for both the natural peptide agonists and the small-molecule agonist compound **1** (Supplementary Fig. 1). We used a Gα-subunit based on a previously described chimeric minimal Gα-construct with altered receptor specificity to allow productive interaction with G_q_-coupled GPCRs^24^. We included residues of the αN helix of Gα_i1_ to obtain a Gα-construct we refer to as mini-G_sqi_. The altered amino-terminus allowed the use of previously described antibody fragment scFv16 that stabilizes the nucleotide-free receptor–G-protein complex^25,26^. To obtain a structure of OX_2_R activated by an endogenous peptide agonist, we purified the receptor in the presence of OxB and assembled a complex with mini-G_sqi_, Gβ_1_γ_2_, and scFv16. Samples were vitrified on electron microscopy grids and the structure was determined by single-particle cryo-EM to a nominal resolution of 3.2 Å (Supplementary Table 1 and Supplementary Fig. 2). The resulting density map was of high quality and allowed modeling of sidechains for most of the amino acids of the complex (Supplementary Fig. 3). Residues N20–M28 at the carboxy-terminus of OxB were well-resolved, while only poor or no density for the remainder of the peptide was observed. We obtained improved density maps at an overall resolution of 3.0 Å for the structure with compound **1** (Fig. 1a) by including a synthetic nanobody, Sb51, for additional conformational stabilization and to aid alignment of particle projections (Supplementary Table 1, and Supplementary Figs. 4 and 5). Sb51 binds to the extracellular surface of OX_2_R contacting residues in extracellular loop (ECL) 2, and part of ECL3 without directly interacting with compound **1** or significantly altering the structure of the small-molecule binding site, suggesting that the compound **1**–receptor interactions are likely unaffected by the presence of the nanobody. Sb51 was omitted, however, for the complex with OxB as it was likely to clash with the much larger peptide agonist or indirectly affect the receptor–peptide interface.

### Overall structures of active-state OX_2_R

**Fig. 2:**
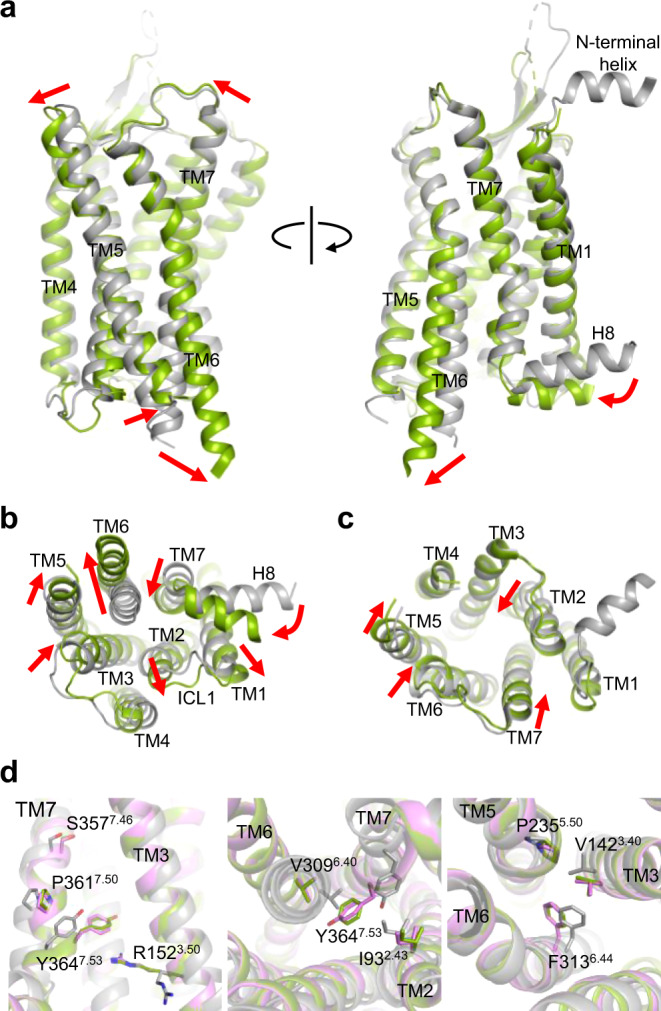
Comparison of inactive-state and active-state OX_2_R. **a** Superposition of OxB-activated (green) and inactive-state OX_2_R (gray; PDB ID 5WQC) viewed from two different angles from within the membrane. Red arrows indicate conformational transitions upon OX_2_R activation. **b**, **c** Intracellular and extracellular view of the superimposed receptor structures, respectively. Residues 192–214 of ECL2 have been removed for clarity. **d** Arrangement of conserved motifs associated with receptor activation: DRY (left), NPxxY (middle), and hydrophobic core triad (right). Structures with OxB (green) and compound **1** (purple) are overlaid with inactive-state OX_2_R (gray).

The structures of OX_2_R bound to OxB and compound **1** are very similar, with root mean square deviations of 0.77 and 0.54 Å, when comparing equivalent α-carbons of OX_2_R and of the entire receptor–G-protein complexes, respectively (Supplementary Fig. 6). The main structural differences are in ECL2 and ECL3 of OX_2_R, regions that contribute to the epitope of Sb51. The structures of the nucleotide-free complexes and the relative orientation of the individual subunits closely resemble previously reported structures of GPCRs bound to G proteins G_i_, G_o_, G_s_, or G_11_ (refs. ^25,27–30^), and are consistent with what has been referred to as a canonical-state complex^31^ (Fig. 1c, d). Comparison of the OxB-bound structure with the structures of inactive-state OX_2_R reveals the conformational changes the receptor undergoes upon agonist binding and activation (Fig. 2a–c). The observed reorganization of transmembrane (TM) helices 5–7 at the cytosolic interface are consistent with the key conformational changes associated with the conserved mechanism of GPCR activation^32,33^, and result in the intracellular half of TM6 swinging outward to allow insertion of the α5 helix at the carboxy-terminus of the Gα-subunit deep into the core of OX_2_R. Furthermore, the arrangements of several structural motifs associated with GPCR activation suggest that the receptor has been visualized in a fully activated state (Fig. 2d). They include the microswitches R152^3.50^ (Ballesteros-Weinstein numbering in superscript^34^), and Y364^7.50^ of the D^3.49^R^3.50^Y^3.51^ motif and the N^7.49^P^7.50^xxY^7.53^ motif, respectively, as well as the hydrophobic core triad I/V^3.40^P^5.50^F^6.44^ (also termed connector region).

The rearrangements on the extracellular side are more subtle and lead to the contraction of the solvent-accessible central cavity of the receptor (Fig. 1c). Of note is that in the structures of antagonist-bound OX_2_R, TM2 is partially unwound and kinked toward TM7 facilitated by conserved P109^2.59^, while in active-state OX_2_R, the helical twist is tightened, causing an inward displacement of residues on either side of the kink. Moreover, enabled by sidechain rearrangements in the hydrophobic core of OX_2_R, TM3 undergoes a minor rotation and moves along the helical axis toward the extracellular side. In addition, the entire helix is slightly shifted toward the center of the helical bundle.

### Peptide binding in the receptor core

**Fig. 3:**
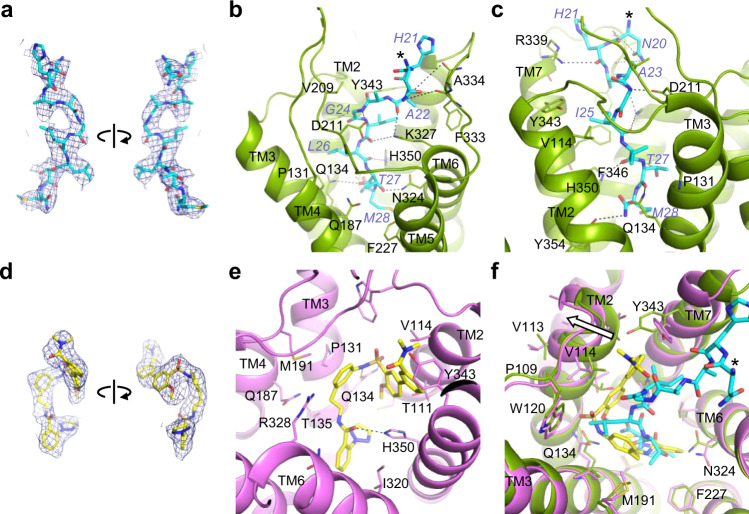
Small-molecule and peptide agonist recognition. **a** Electron density map (blue mesh) around residues N20–M28 of OxB (cyan) viewed from two angles. **b**, **c** Two different views of the detailed interactions of OxB and OX_2_R (green). **d** Electron density map (blue mesh) around compound **1** (yellow) viewed from two angles. **e** Detailed interactions of compound **1** with OX_2_R (purple). **f** Overlay of the binding sites with OxB and compound **1**. Residues 192–214 of ECL2 have been removed for clarity. The asterisks in **a**, **c**, and **f** indicate the position of the mainchain nitrogen of N20 of OxB.

The 28 amino acid neuropeptide OxB is a potent orexin agonist with modest selectivity for OX_2_R (Fig. 1b)^3^. Our cryo-EM map showed strong density only for the carboxy-terminal portion of OxB (N20–M28). This segment adopts an extended conformation and reaches far into the core of OX_2_R, contacting all TM helices except TM1, as well as residues in ECL2 and ECL3 through an extensive interface of hydrophobic and polar interactions (Fig. 3a–c and Supplementary Table 2). This extended conformation was unanticipated since in solution both OxA and OxB fold into two α-helices oriented approximately perpendicular to each other^35,36^, and molecular docking of OxA and OxB to models of both receptors suggested plausible binding poses with α-helical carboxy-terminal portions^37^. In contrast, the natural peptide agonists of the endothelin ET_B_ receptor and neurotensin NTS_1_ receptor, both GPCRs of the same subfamily as OX_2_R, are disordered in isolation and only become conformationally restricted upon binding to their cognate receptors^38–42^. Comparison with the peptide-bound ET_B_ and NTS_1_ receptors shows that the carboxy-terminal end of OxB overlays well with those of endothelin-1 and endothelin-3 (ET-1 and ET-3, respectively), while that of neurotensin binds ~8 Å higher up in the binding pocket (Supplementary Fig. 7).

OxB residues G24–M28 are sequestered from the extracellular milieu, adopting high shape complementary with the bottom of the binding pocket. Individual mutation of any of these residues to alanine causes a decrease in potency of two to three orders of magnitude, confirming the importance of this tight interface^43^. Of note are contacts mediated by the sidechains of I25 and carboxy-terminal M28. The former is sandwiched between the sidechains of Y343^7.32^ and F346^7.35^, while the latter reaches into the narrow bottom of the pocket, interacting with a lipophilic patch formed by F227^5.42^, T231^5.46^, and I320^6.51^. In addition, there are several hydrogen bond interactions in this region, one between N324^6.55^ and T27, another between the backbone carbonyl of T27 and the Q134^3.32^, and a third, possibly water-mediated, between H350^7.39^ and the backbone carbonyl of L26. The amidated carboxy-terminus of OxB is within hydrogen bonding distance of the hydroxy group of Y354^7.43^. Similar polar interactions at the base of the binding pocket between an agonist peptide and residues at positions 3.32 and 7.43 have been observed for the µ- and δ-opioid receptors^25,44^. In the ET_B_ receptor, Q181^3.32^ forms hydrogen bonds with the backbones of ET-1 and ET-3 in much the same way as observed in the OX_2_R–OxB complex^39,40^. The pocket widens toward the extracellular side, exposing residues N20–A23 of OxB to the surrounding milieu. Several polar interactions with the mainchain and sidechains of OxB form the interface with OX_2_R in this region. The sequence of the carboxy-terminal segment of OxB differs from that of OxA only at the terminal residue (L33 in OxA, M28 in OxB; Supplementary Fig. 3a). It is therefore likely that the observed receptor–peptide interactions made in this region are conserved across the two endogenous agonists.

### Receptor–peptide interactions at the extracellular surface

**Fig. 4:**
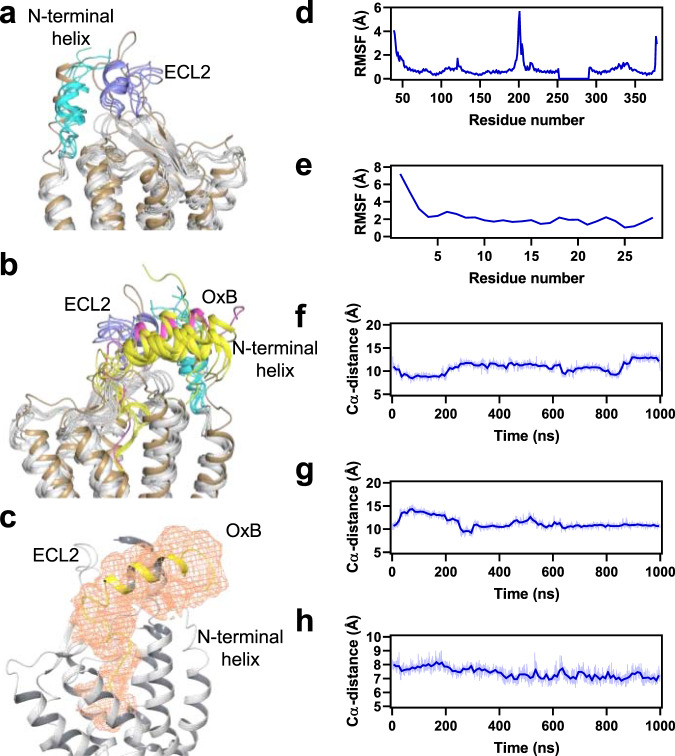
Molecular dynamics simulations. **a**, **b** Representative frames from a 1000-ns MD trajectory overlaid with the model of OX_2_R bound to full-length OxB (sand). OX_2_R and OxB ensembles from the simulation are colored gray and yellow, respectively. The amino-terminal α-helix and ECL2 of OX_2_R are highlighted in cyan and light blue, respectively. Note, in **a**, OxB was omitted for clarity. **c** MD-derived density map (orange mesh) of OxB defining the space occupied by the peptide during the simulations, showing that the amino-terminal portion of OxB remains α-helical, but is more flexible than the extended carboxy-terminal portion buried in the OX_2_R core. **d**, **e** Root mean square fluctuation (RMSF) values of α-carbons of OX_2_R and OxB, respectively, during a representative simulation. **f**–**h** Distances between α-carbons of pairs of residues in OxB and OX_2_R monitored over the course of the same representative simulation. Residue pairs are: Q12(OxB)–F197 (ECL2 of OX_2_R), Q12(OxB)–E46 (amino-terminal helix of OX_2_R), and I25(OxB)–F346^7.35^ (core of OX_2_R), respectively. A central moving average (window length: 10 ns) of the α-carbon distance for each pair is indicated by a dark blue line. Source data are provided as a Source Data file.

Our cryo-EM map contains two less well-defined density features protruding into the extracellular space that are absent in the map of the complex with compound **1** (Supplementary Fig. 8): one is continuous with the amino-terminal end of TM1 and likely accounts for a short α-helix observed in structures of antagonist-bound OX_1_R and OX_2_R^21–23^, albeit in varying orientations, indicating flexibility in the absence of a peptide agonist. In proximity is the second feature that connects to the stronger density describing the carboxy-terminal segment of OxB. Its shape and location are consistent with an α-helical amino-terminal portion of the peptide. While we excluded both structural elements from our final model of the OX_2_R–G-protein complex, we hypothesized that the amino-terminal segment of OxB forms a tripartite interaction with the amino-terminal α-helix and ECL2 of OX_2_R. The involvement of these parts of the receptor in peptide-mediated activation has been demonstrated by mutagenesis^21,45^. Guided by the cryo-EM map, we constructed a model of OX_2_R bound to full-length OxB comprising residues of the amino-terminal α-helix and a complete ECL2 (Supplementary Fig. 8). To probe the stability of the receptor–peptide interface in the extracellular region, this model was subjected to all-atom molecular dynamics (MD) simulations (Fig. 4). We performed four independent 1000-ns MD simulations for a combined total of 4 μs of simulation time. The helical structures of the amino-terminal portion of OxB and the amino-terminal α-helix of OX_2_R, as well as the β-sheet in ECL2 were found to be stable throughout each microsecond-scale MD trajectory. Some flexibility was observed for the distal region of ECL2, where in two trajectories formation of a short α-helix was observed. However, the OX_2_R–OxB interface remained intact throughout all simulations with the peptide in contact with both ECL2 and the amino-terminal α-helix of OX_2_R. Together, the cryo-EM map and the MD simulations suggest that these extracellular regions of OX_2_R engage in agonist peptide binding.

### Small-molecule agonist recognition

We have recently discovered diarylsulfonamide compound **1**, a potent and selective OX_2_R agonist (Fig. 1a, b). With a local resolution of 3 Å, the cryo-EM map was of excellent quality in the OX_2_R core, featuring well-defined density for a single molecule of compound **1** in the orthosteric pocket (Fig. 3d, e and Supplementary Fig. 5). The ligand is kinked around a central sulfonamide, positioning the two flanking phenyl rings approximately perpendicular to one another. This core interacts extensively with OX_2_R: the sulfonamide forms a bidentate hydrogen bond interaction with Q134^3.32^, while the adjacent phenyl rings contact a lipophilic patch formed by P131^3.29^, M191^4.64^, T111^2.61^, and V114^2.64^. The two ends of the compound project into distinct regions. The amide-linked phenyltriazole inserts into the hydrophobic bottom of the pocket that accommodates carboxy-terminal M28 of OxB in peptide-bound OX_2_R. It makes hydrophobic contacts to C107^2.57^, T135^3.33^, V138^3.36^, I320^6.51^_,_ and N324^6.55^ and binds through π–π interactions to F227^5.42^. The amide carbonyl of the linker forms a hydrogen bond with H350^7.39^, whose sidechain also contacts the terminal 1,2,3-triazole moiety and the distal phenyl ring of the core, thereby stabilizing the kinked conformation of compound **1**. The other end of the agonist extends toward the extracellular side, a region occupied by I25 of OxB in the OX_2_R–OxB complex. However, facilitated by conserved P109^2.59^, the last two turns of TM2 are shifted outward in compound **1**-bound OX_2_R, enlarging the subpocket between TM2 and TM7 to accommodate the substituted biphenyl fragment of the ligand. In addition, Y343^7.23^ assumes a different sidechain rotamer to interact with the terminal *N*,*N*-dimethylbenzamide (Fig. 3f). The observed ligand conformation and receptor–ligand interactions are unrelated to those proposed for a similar non-peptide OX_2_R agonist based on molecular docking into models derived from a structure of antagonist-bound OX_2_R^16,46^, underscoring the need for experimental structures to guide drug design.

### Comparison with antagonist binding

**Fig. 5:**
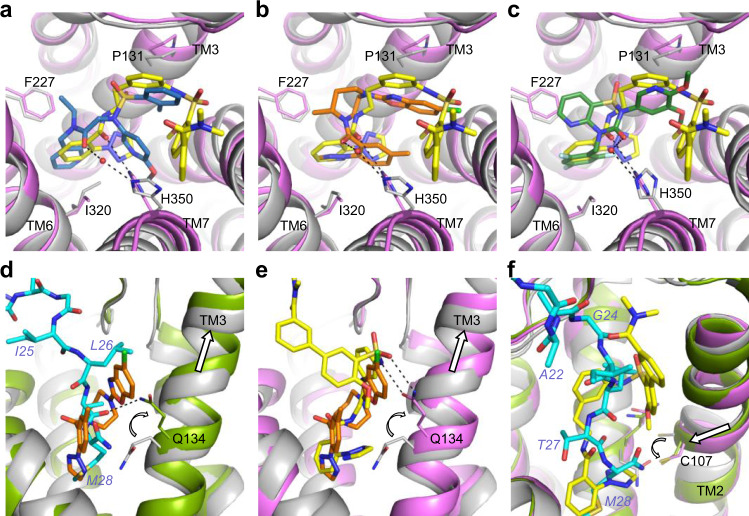
Comparison of agonist and antagonist binding. Superpositions of OX_2_R in inactive (gray) and active (green and purple) conformations. **a**–**c** Comparison of the binding mode of compound **1** (yellow) and those of the antagonists EMPA (blue; PDB ID 5WQC), suvorexant (orange, 4S0V), and HTL6641 (dark green; 6TPN), respectively. Water molecules mediating interactions with the sidechain of H350^7.39^ in the antagonist-bound complexes are rendered as red spheres. **d**–**f** Conformational changes (white arrows) in TM2, TM3, Q134^3.32^, and C107^2.56^ upon activation by OxB (cyan) and compound **1** are highlighted by white arrows.

Superposition of our structures of agonist-bound OX_2_R with those of the inactive receptor bound to the antagonists suvorexant^20^, HTL6641 (ref. ^23^), or EMPA^22^ reveals that all three antagonists occupy the bottom-most region of the central cavity, overlapping with the last three residues of OxB and the portion of compound **1** containing the sulfonamide and the amide-linked phenyltriazole. Compound **1** and all three antagonists place similar chemical groups in three distinct locations (Fig. 5a–c): (i) an aromatic ring is anchored at the bottom between the sidechains of F227^5.43^ and I320^6.51^. (ii) An amide carbonyl is placed in the center of the pocket, interacting with H350^7.39^. Notably, this contact is water-mediated in the case of all three antagonists^20,22,23^, whereas in active-state OX_2_R, owing to conformational changes in TM7, H350^7.39^ sits lower and more central, allowing direct interaction with the amide linker in compound **1**. (iii) A second aromatic moiety is positioned further up overlapping with the location of the sidechain of L26 of OxB. Although for both compound **1** and all three antagonists, these groups interact with the lipophilic patch around P131^3.29^, the phenyl ring of the compound **1** diarylsulfonamide core is located further toward the extracellular side. This is noteworthy as it leaves space for Q134^3.32^ to adopt an extended conformation and to make the observed bidentate interaction with the sulfonamide of the agonist. Similarly, the position of L26 of OxB allows Q134^3.32^ to extend and form a hydrogen bond with the OxB backbone. It is the position and sidechain rotamer of this residue that constitutes the most substantial difference in the binding sites of inactive-state and active-state OX_2_R (Fig. 5d, e and Supplementary Fig. 9). Our cryo-EM maps unambiguously show that in both structures of agonist-bound OX_2_R, the sidechain of Q134^3.32^ is extended and projects upward, i.e., toward the extracellular side (Supplementary Figs. 3f and 5f). In addition, this configuration of Q134^3.32^ and its interaction with OxB remained stable in our microsecond-scale MD simulations (Supplementary Fig. 10). In contrast, this sidechain is positioned further down and points downward toward the cytoplasm, when OX_2_R is bound to any of the antagonists. A similar arrangement of the equivalent residue Q126^3.32^ is observed in all available structures of OX_1_R captured in an inactive state (Supplementary Fig. 11)^21,23^. High-resolution structures of both receptors reveal that this sidechain conformation is stabilized by a hydrogen bond to a tyrosine at position 7.43, and through interactions with a network of water molecules and polar sidechains, which in OX_1_R also includes a sodium ion^22^.

## Discussion

The cryo-EM structures of active-state OX_2_R bound to OxB and compound **1** reveal the molecular details of peptide and small-molecule agonist binding. Both OxB and compound **1** are selective orexin agonists with one and four orders of magnitude higher potency at OX_2_R compared to OX_1_R, respectively (Fig. 1b). With active-state structures of only one of the two receptors and only with selective agonists at hand, additional structural and functional data will be required to obtain a detailed understanding of the molecular details underlying receptor subtype selectivity. However, the selectivity of OxB and that of compound **1** likely have distinct molecular bases. The sequence of the last nine amino acids of OxB only differs from that of nonselective OxA at the terminal residue (Met in OxB and Leu in OxA). Therefore, subtype selectivity likely arises from interactions of the amino-terminal portions of the two peptides, which differ both in sequence and structure^35,36^, with residues at the extracellular surface of the receptors, where the sequences of OX_1_R and OX_2_R diverge. In contrast, the binding site of compound **1** is entirely located in the highly conserved core of the receptor, where OX_2_R only differs from OX_1_R at positions 2.61 (T111^2.61^ in OX_2_R; S103^2.61^ in OX_1_R) and 3.33 (T135^3.33^ in OX_2_R; A127^3.33^ in OX_1_R). The resulting differences in shape and electrostatics of the small-molecule agonist binding sites of the two receptors are likely subtle, making it challenging to understand the basis for receptor subtype selectivity, in particular in the absence of structures of active-state OX_1_R. Since compound **1** does not make any direct polar contacts with T111^2.61^ or T135^3.33^, it is tempting to speculate that water-mediated interactions and/or rearrangements of water networks in the binding pocket have a key role in the observed selectivity for OX_2_R akin to what has recently been proposed for the OX_2_R-selective antagonist EMPA based on high-resolution crystal structures of EMPA-bound OX_1_R and OX_2_R^23^. Additional structures of active-state OX_1_R and OX_2_R bound to subtype-specific small-molecule agonists—preferably at resolutions at which water molecules can be resolved—will be required to elucidate the molecular bases of small-molecule agonist selectivity.

The structures presented in this study suggest that interactions in the core of the receptor are sufficient to elicit the conformational changes in OX_2_R required for receptor activation. OxB induces these rearrangements through an extended carboxy-terminal segment inserted deep into the orthosteric pocket. This is in agreement with previous observations that OxA truncated from its amino-terminus down to a segment that is highly conserved between the two endogenous neuropeptides contains the residues critical for functional potency on OX_1_R^47^. Our experimental and computational results also demonstrate the presence of contacts between the amino-terminal portion of OxB and extracellular regions of the receptor consistent with a model proposed based on mutagenesis studies of OX_1_R, in which the peptide agonist binds to the receptor through a polytopic interface^21^. In line with the weaker density observed in the cryo-EM map, our MD simulations reveal increased flexibility of the receptor–peptide interface in the extracellular region compared to that in the OX_2_R core. The amino-terminal portion of OxB, while not directly involved in activation, may have a role in initial binding of the peptide and may function as “address” component of a classic “message-address” system^48^, in which the peptide’s carboxy-terminal segment carries the “message”. This concept has also been proposed for peptide agonist binding to other receptors, including opioid, nociceptin, and, endothelin receptors^49–52^.

**Fig. 6:**
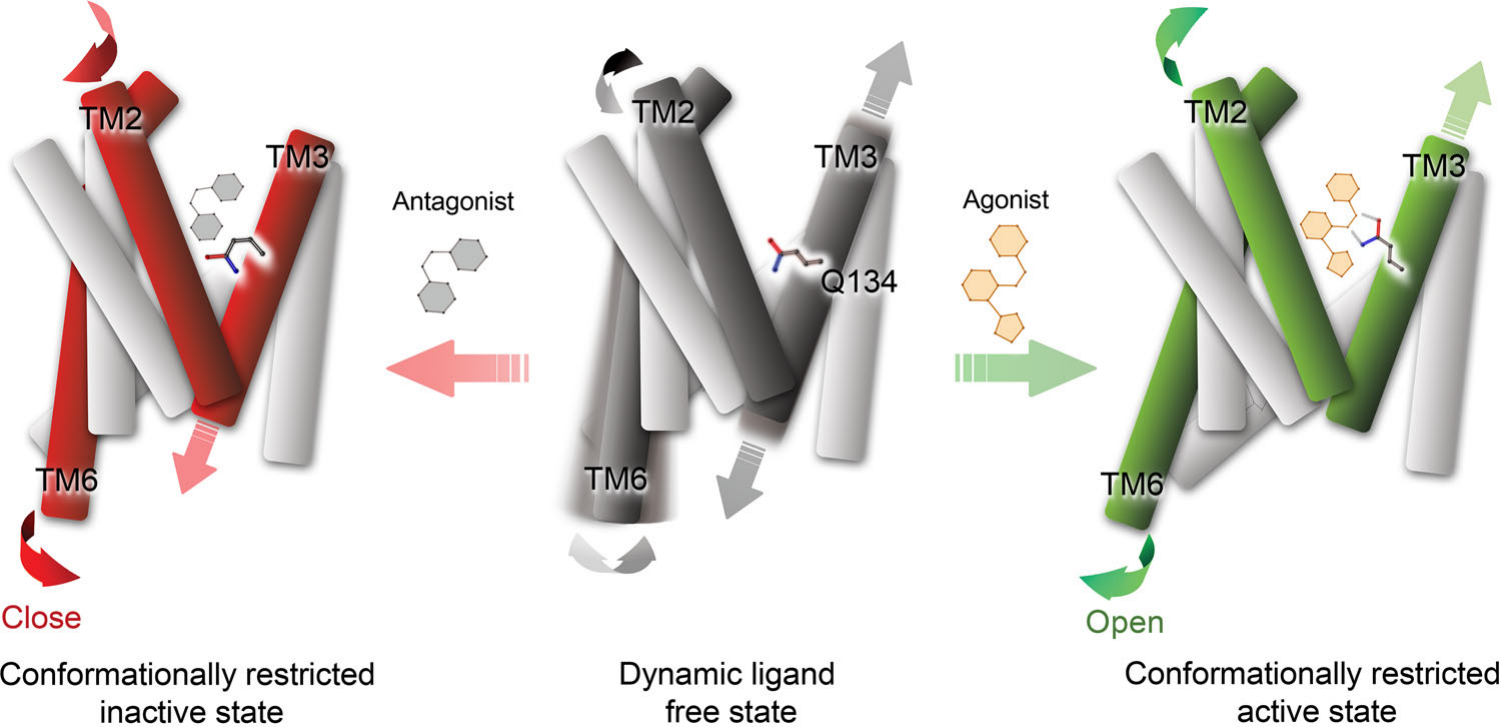
Mechanism of OX_2_R activation and inactivation. Schematic representation of OX_2_R in ligand-free (middle), antagonist-bound (left), and agonist-bound (right) states. The sidechain of mechanistically important Q134^3.32^ is shown in stick representation. Conformational flexibility in TM2, TM3, and TM6 in the ligand-free state is indicated with gray arrows, while conformational changes upon agonist and antagonist binding are highlighted by green and red arrows, respectively.

Based on the key structural differences between agonist- and antagonist-bound OX_2_R, we propose a common mechanism of activation by the endogenous peptide agonists, as well as the small-molecule agonist compound **1** (Fig. 6). This mechanism also rationalizes how antagonists of the three classes discussed above keep the receptor in an inactive conformation. GPCRs are dynamic proteins that transition between fully inactive, fully active, and intermediate state conformational ensembles even in the absence of a ligand^53,54^. Binding of an agonist or antagonist shifts this dynamic equilibrium toward an active or inactive conformation by stabilizing the respective arrangement of key structural elements. The outward swing of TM6, essential for the formation of the receptor–G-protein complex, is coupled to the upward movement of TM3 through rearrangements in the connector region^53,55^. Our structures reveal that both OxB and compound **1** stabilize the upward displacement of TM3 in two ways: (i) direct stabilization through hydrogen bond interaction with the upward-shifted and extended sidechain of Q134^3.32^ and (ii) indirect stabilization by filling the space vacated by the relocated Q134^3.32^. For OxB, this is accomplished by part of the sidechain and the carboxy-terminal amide group of M28, while in the case of compound **1** it is the terminal 1,2,3-triazole group that is inserted. The upward movement of TM3 is accompanied by an inward shift of TM2. Consequently, C107^2.56^ moves adjacent to the 1,2,3-triazole of compound **1** and the carboxy-terminal amide group of OxB, respectively, effectively forming a wedge that prevents TM3 from sliding down into the position observed in inactive-state OX_2_R and Q134^3.32^ from adopting the downward-facing sidechain rotamer (Fig. 5f). In contrast, the antagonists act in the opposite way, stabilizing inactive-state OX_2_R by placing a bulky substituent in a position that would clash with the upward-shifted and extended Q134^3.32^, and by leaving room for the downward-facing rotamer of this sidechain found in inactive-state OX_2_R. Whether the mechanism proposed here extends to other classes of orexin agonists remains to be elucidated, but it is consistent with all available structures of inactive-state OX_1_R and OX_2_R determined with antagonists of diverse chemotypes. Despite involving a common set of structural rearrangements at the intracellular coupling interface, activation of different GPCRs follows different mechanisms, reflecting the chemical diversity of their agonists^53,56^. To our knowledge, there is no precedent for such a central role of the sidechain at position 3.32 facilitating the transition to an activated state of a GPCR, as proposed here for the orexin receptors. The identification of the molecular basis of activation provides critical information for structure-based drug discovery of orexin agonists for the treatment of NT1 and other hypersomnia disorders.

## Methods

### Synthesis of compound 1

The synthesis of compound **1** was accomplished in four steps from commercially available reagents. An EDC/HOAt-mediated amide bond formation between 2-(2H-1,2,3-triazol-2-yl)benzoic acid and 2-(3-nitrophenyl)ethanamine hydrochloride proceeded in nearly quantitative yield to afford an aryl nitro intermediate. The nitro group was reduced under a hydrogen atmosphere with Pd/C catalyst to reveal an aniline intermediate, *N*-(3-aminophenethyl)-2-(2H-1,2,3-triazol-2-yl)benzamide. A sulfonamide was formed by reaction of the aniline intermediate with 5-bromo-2-methoxybenzenesulfonyl chloride using pyridine as the base. The resulting aryl bromide was subjected to a Pd-catalyzed Suzuki reaction with 3-(dimethylcarbamoyl)phenylboronic acid to give 3′-(*N*-(3-(2-(2-(2H-1,2,3-triazol-2-yl)benzamido)ethyl)phenyl)sulfamoyl)-4′-methoxy-*N*,*N*-dimethyl-[1,1′-biphenyl]-3-carboxamide, compound **1** in an overall 73% isolated yield with 98.9% purity.

### Expression and purification of OX_2_R

Human OX_2_R truncated after residue R389, with part of ICL3 (residues V261–A291) deleted, and carrying a Flag epitope tag and a deca-histidine tag at its amino and carboxy termini, respectively, was expressed in transiently transfected mammalian cells in suspension using the Gibco Expi293 Expression System (Thermo Fisher). Cells were seeded at 1.25 × 10^6^ viable cells/ml into 5 L Optimum Growth Flasks (Thomson Instrument Company), grown with shaking at 37 °C overnight, and transfected according to the manufacturer’s instructions. Expression enhancers were added at 16–18 h post transfection and the temperature was decreased to 30 °C. Cells were harvested 46–50 h post transfection and stored at −80 °C until further use. All purification steps were carried out at 4 °C. All purification buffers were supplemented with either 20 μM compound **1** or 2 μM OxB (Phoenix Pharmaceuticals). Cells were thawed and lysed using a Dounce homogenizer in buffer containing 50 mM Tris-HCl pH 7.5, 10% (v/v) glycerol, 50 mM ammonium acetate, and supplemented with 1 mg/ml iodoacetamide, cOmplete EDTA-free protease inhibitors (Roche), and 100 units/ml Benzonase nuclease (Sigma-Aldrich). The lysate was incubated with ligand for 1 h prior to solubilization. Cells were then combined with 1.0% (w/v) *n*-dodecyl-β-D-maltopyranoside (DDM), and 0.1% (w/v) cholesteryl hemisuccinate (CHS) and gently mixed for 1 h. Insoluble material was removed by ultracentrifugation at 100,000 × *g* for 35 min. Receptors were immobilized by batch binding to 12.5 ml anti-Flag M2 immunoaffinity resin (Sigma-Aldrich) for 1 h. The resin was packed into a column and washed with ten column volumes (CV) of buffer containing 50 mM Tris-HCl pH 7.5, 50 mM ammonium acetate, 0.05% (w/v) DDM, and 0.005% (w/v) CHS. Bound material was eluted with buffer supplemented with 0.2 mg/ml Flag peptide. The receptor was bound to 1 ml Talon immobilized metal-chelate chromatography (IMAC) resin (Takara Bio) overnight. The resin was washed with 10 CV of buffer containing 50 mM Tris-HCl pH 7.5, 50 mM ammonium acetate, 0.05% (w/v) DDM, and 0.005% (w/v) CHS and OX_2_R was eluted with 300 mM imidazole.

### Synthetic nanobody selection

Synthetic nanobodies were selected against OX_2_R following procedures described previously^57^. Engineered OX_2_R, carrying an additional amino-terminal Avi-tag, was expressed and purified in the presence of 50 µM small-molecule agonist as described above and biotinylated using BirA biotin-protein ligase (Avidity LLC) according to the manufacturer’s instructions. Binders to the immobilized receptor were selected from the three synthetic nanobody libraries, constructed by Zimmermann and coworkers^57^, by ribosome display followed by phage display as described^57^. Two rounds of selection were performed in the presence of 50 µM small-molecule agonist. In a third round, selections were competed with an excess of unbiotinylated OX_2_R in the absence of agonist to enrich high-affinity binders against agonist-bound OX_2_R. A total of 570 clones were tested by ELISA as described^57^, using an unrelated membrane protein to eliminate nonspecific binders. Sb51 was identified from a set of 57 unique hits based on expression level, sample homogeneity after purification, and affinity for agonist-bound OX_2_R (Supplementary Data 1). The dissociation constant *K*_D_ for the binding of Sb51 to agonist-bound OX_2_R was ~0.6 nM as determined by grating-coupled interferometry on a WAVE system (Creoptix), using OX_2_R immobilized via the biotinylated Avi-tag on a neutravidin-coated WAVEchip 4PCP (Creoptix).

### Expression and purification of G-protein subunits

Mini-G_sqi_ was constructed by adding an octa-histidine tag followed by a Tobacco Etch virus (TEV) protease site and residues 4–18 of human Gα_i1_ to the amino-terminus of mini-G_s/q_71, a chimeric minimal G protein described previously^24^. Mini-G_sqi_ and Gβ_1_γ_2_-dimer were expressed and purified following methods described earlier^58^. Briefly, mini-G_sqi_ was expressed in *Escherichia coli* and purified by IMAC. The purification tag was removed using TEV protease followed by IMAC. The protein was further purified by size-exlcusion chromatography (SEC) in 10 mM HEPES-NaOH pH 7.5, 10% (v/v) glycerol, 100 mM NaCl, 1 mM MgCl_2_, 1 µM guanosine 5′-diphosphate (GDP), and 100 µM tris(2-carboxyethyl)phosphine (TCEP), concentrated to 40–45 mg/ml, flash cooled in liquid nitrogen, and stored at −80 °C until further use. Non-lipidated Gβ_1_γ_2_-dimer was expressed in *Trichoplusia ni* insect cells (Expression Systems) using the titerless infected-cells preservation and scale-up method^59^. Cells were coinfected with two baculoviruses carrying human Gβ_1_ with an amino-terminal octa-histidine tag and human Gγ2 containing mutation C68S, respectively. Each baculovirus was added at an estimated multiplicity of infection of 5. The dimer was purified by IMAC followed by anion exchange and SEC in 10 mM HEPES-NaOH pH 7.5, 10 % (v/v) glycerol, 100 mM NaCl, 100 µM TCEP, concentrated to 10–15 mg/ml, flash cooled in liquid nitrogen, and stored at −80 °C until further use.

### Expression and purification of scFv16 and Sb51

Both antibody fragment scFv16 (ref. ^26^) and synthetic nanobody Sb51 were overexpressed in the periplasm of *E. coli* C41(DE3) as fusion proteins with *E. coli* maltose-binding protein (MBP). Their amino-termini were fused to the carboxy-terminus of MBP, carrying an amino-terminal deca-histidine tag, via a linker containing a TEV protease site. Bacterial cultures were grown in Terrific Broth supplemented with 0.5% (w/v) glucose and 5 mM MgSO_4_ at 30 °C to an optical density at 600 nm of 0.8–1.0. Protein expression was induced by the addition of isopropyl-β-D-thiogalactoside to a final concentration of 50 µM. The temperature was lowered to 22 °C and cultures grown for additional 18–20 h. All subsequent steps were carried out at 4 °C. For purification of nanobody Sb51, bacterial cells were resuspended in buffer containing 50 mM Tris-HCl pH 7.5, 150 mM NaCl, and 15 mM imidazole-HCl pH 7.5, supplemented with cOmplete EDTA-free protease inhibitors, and lysed by ultrasonication. After removal of insoluble material by centrifugation, the fusion protein was bound to NiNTA Superflow metal-affinity resin (Qiagen). The resin was washed extensively with 60 mM imidazole prior to elution of the protein with 300 mM imidazole. The fusion tag was cleaved off with TEV protease in a 1:25 (w/w) ratio of protease to nanobody and incubation at 4 °C for 16 h. Concomitantly, the buffer was exchanged to 10 mM Tris-HCl pH 7.5 and 150 mM NaCl by dialysis before the mixture was passed over a column containing NiNTA Superflow affinity resin. The protein was diluted tenfold with 20 mM HEPES-NaOH pH 7.0, bound to a 5 ml HiTrap SP HP cation exchange column (GE Healthcare), and eluted in a linear gradient of 0–500 mM NaCl over 60 CV. Fractions containing nanobody Sb51 were dialyzed against a buffer containing 10 mM Tris-HCl pH 7.5, 10% (v/v) glycerol, and 150 mM NaCl. The protein was concentrated to 10–15 mg/ml, flash cooled in liquid nitrogen, and stored −80 °C until further use. For purification of scFv16, bacterial cells were resuspended in 50 mM Tris-HCl pH 7.5, 150 mM NaCl, and 25 mM imidazole-HCl pH 7.5, supplemented with cOmplete EDTA-free protease inhibitors and lysed by ultrasonication. The soluble fraction was passed over a column containing NiNTA Superflow resin. The column was washed with 60 mM imidazole and scFv16 was eluted with 300 mM imidazole. The fusion tag was removed by digestion with TEV protease at a ratio of 1:40 (w/w), protease to scFv16, and simultaneous dialysis against 20 mM Tris-HCl pH 7.5 and 0.15 M NaCl at 4 °C for 16 h. Uncleaved material was separated by rebinding to NiNTA Superflow. Remaining contaminants were eliminated on a Superdex 200 Increase 10/300 GL SEC column (GE Healthcare) equilibrated in 10 mM HEPES pH 7.5, 10% (v/v) glycerol, and 100 mM NaCl. The protein was concentrated to 8–10 mg/ml, flashed cooled in liquid nitrogen, and stored at −80 °C until further use.

### Reconstitution of OX_2_R–G-protein complex

Purified OX_2_R was concentrated and complexed with mini-G_sqi_, Gβ_1_γ_2_-dimer, and scFv16 in a 1:1.5:1.5:2 molar ratio by overnight incubation at 4 °C in the presence of 20 μM compound **1** or 25 μM OxB, and six units apyrase (Sigma-Aldrich) to remove GDP released upon complex formation. For the complex with compound **1**, two molar equivalents of nanobody Sb51 were included. In parallel, the receptor was deglycosylated using 500 μg PNGaseF. The mixture was further purified by SEC using a Superdex 200 Increase 10/300 GL column equilibrated in buffer containing 25 mM Tris-HCl pH 7.6, 50 mM ammonium acetate, 0.02% (w/v) DDM, 0.002% (w/v) CHS, 0.5 mM EDTA, and 20 μM compound **1** or 2 μM OxB. Fractions containing the complex were pooled and concentrated to ~5 mg/ml. Protein concentration was determined using a calculated extinction coefficient at 280 nm (*ε*_280_, calc = 306,580 ml mg^−1^ cm^−1^ for the complex with compound **1**; *ε*_280_, calc = 282,045 ml mg^−1^ cm^−1^ for the complex with OxB).

### Cryo-EM grid preparation and data collection

Aliquots (3 μl) of purified complex were applied to glow-discharged (20 s on carbon side) C-flat 20 nm thickness holey carbon-on-gold grids (300 mesh, R1.2/1.3). The grids were blotted for 3 s at 95% humidity and plunge-frozen into liquid ethane using a Vitrobot Mark IV (Thermo Fisher Scientific). Grids were imaged on a 300 keV Titan Krios cryo-electron microscope (Thermo Fisher Scientific) equipped with an energy filter (Gatan GIF BioQuantum) and a post-GIF Gatan K3 Summit direct electron detector. Images were taken on the K3 camera in dose-fractionation mode at a calibrated magnification of 59,524, corresponding to 0.84 Å per physical pixel (0.42 Å per super-resolution pixel). The dose fractionation on the specimen was set to be 1.0625 electrons per Å^2^ per frame and the total number of frames was 40, resulting in a total dose of 42.5 electrons per Å^2^. An energy slit with a width of 20 eV was used during data collection. Fully automated data collection was carried out using Latitude in Gatan Imaging Suite (Gatan Inc.) with a nominal defocus range set from −0.8 to −2.5 μm. Image Shift was used with nine exposure groups per stage shift to improve the throughput of data collection. A total of 38,810 and 17,956 movies were collected for the samples with OxB and compound **1**, respectively. For data collection is summarized in Supplementary Table 1, and Supplementary Figs. 2 and 4.

### Cryo-EM data processing

The cryoSPARC Live application of cryoSPARC v. 2 (Structura Biotechnology) was used to streamline the movie processing, CTF estimation, particle picking, and 2D classification. Preprocessing involved anisotropic motion correction^60^ and local CTF estimation. The data were curated by keeping only data with better than 6 Å determined by CTF fit resolution. Particle picking started with blob picking ~150 Å in diameter. Once a small set of particles was extracted and 2D class averages were obtained, the good 2D classes were used as templates for picking on the entire dataset. From the ~14 million (OxB) and ~7.5 million (compound **1**) particles initially picked, ~2.9 million and ~1.9 million particles were kept from the good 2D classes, respectively. Then ab initio 3D reconstruction was carried out in cryoSPARC v. 2 (ref. ^61^) and one out of three classes was giving the reconstruction of the designed complex from ~0.8 million and ~1.1 million particles for OxB and compound **1**, respectively. Homogenous refinement coupled with nonuniform and global CTF refinements gave the final 3D reconstructions at 3.2 Å (OxB) and 3.0 Å (compound **1**) resolution, respectively (0.143 gold-standard FSC with correction of masking effects^62^. Local resolution estimates, shown in Supplementary Figs. 2 and 4, were performed in cryoSPARC v. 2. For data processing, see Supplementary Table 1, and Supplementary Figs. 2 and 4.

### Model building and refinement

Starting models for OX_2_R, mini-G_sqi_, Gβ_1_γ_2_, and scFv16 were based on Protein Data Bank (PDB) entries 4S0V, 5G53, 3SN6, and 6DDE, respectively. For an initial model of nanobody Sb51, a homology model based on PDB entry 3K1K was constructed using Maestro (Schrödinger). For the complex with compound **1**, its five subunits were placed into the density map by rigid-body fitting. The fit of the intracellular half of TM6, Helix8, and ECL2 of OX_2_R, as well as the α5 helix of mini-G_sqi_ was manually adjusted in Coot^63^. Missing residues of the αN helix of mini-G_sqi_ were manually fitted and sequences adjusted where required. The resulting initial model was further refined in iterative rounds of manual modifications in Coot and real-space refinement using the Phenix software package^64^. Coordinates and restraints for compound **1** were generated using Grade (Global Phasing Ltd.), and the ligand was manually fitted into the density using real-space refinement in Coot and further refined using Phenix. The refined model for the complex with compound **1** was used as starting point for the refinement of the structure with OxB, using a similar iterative model building and refinement strategy. Model quality was assessed using Molprobity^65^ as implemented in Phenix. Map and model statistics are detailed in Supplementary Table 1. Even though present in the OX_2_R construct no or poor density was observed for residues M1 to L48 (complex with OxB) or M1 to E54 (complex with compound **1**) preceding TM1, residues C252 to S260 and 290 of ICL3, and residues beyond A378 in Helix8. Due to the absence of Sb51, the complex with OxB additionally lacked density for residues P198 to L206 of ECL2. These regions were therefore left unmodeled. For the same reason, the linker replacing the Gα_s_ helical domain and flanking residue stretches R61 to H64 and T206 to G208 of mini-G_sqi_ (Gα_s_ numbering), residues M1 to N4 and E63 to L71 at the termini of Gγ2, and most residues of the interdomain linker of scFv16 were not included in the final model. In addition, several predominantly surface-exposed sidechains throughout the complex were truncated to their β-carbon atoms due to lack of density. Molecular representations were generated using PyMol (Schrödinger) and Chimera^66^.

### Radioligand-binding assay

Cell membranes from a Chinese hamster ovary (CHO) cell line stably expressing wild-type OX_2_R and from transiently transfected Expi293 cells (Thermo Fisher) overexpressing engineered OX_2_R were incubated at room temperature for 6 h with ^125^I-OxA (PerkinElmer) in assay buffer (25 mM HEPES-NaOH pH 7.4, 2.5 mM CaCl_2_, 1.25 mM MgCl_2_, and 0.175% (w/v) CHAPS) in a total volume of 200 µl. Unbound ligand was removed by rapid filtration through GF/C glass fiber filters and 3 × 3 ml washes with 50 mM Tris-HCl pH 7.4, 200 mM NaCl, and 0.2% (w/v) CHAPS. Bound radioactivity was measured through liquid scintillation using EcoLume Liquid Scintillation Cocktail (MP Biomedicals) and detected using a Tri-Carb liquid scintillation counter. Saturation studies were carried out by incubating membranes (20 μg total protein/well) with a range of concentrations of ^125^I-OxA (0.002–8 nM). For competition studies, membranes (20 μg total protein/well) were incubated with a range of concentrations of compound **1** and OxB (100 µM–0.02 nM and 1 µM–0.0002 nM, respectively) and with ^125^I-OxA at 0.005 nM. Data were analyzed using GraphPad Prism (GraphPad Software, Inc.).

### Inositol monophosphate accumulation assay

Agonist-dependent inositol monophosphate (IP1) accumulation was measured using the IP-One G_q_ kit (Cisbio), a cell-based homogeneous time-resolved fluorescence assay, according to the manufacturer’s instructions. Briefly, cryo-preserved CHO cells stably expressing human OX_1_R and human OX_2_R were thawed, and resuspended in IMDM medium (Gibco) at densities of 2.0 × 10^5^ cells/ml and 4.0 ×10^5^ cells/ml, respectively. Cells were seeded into 384-well plates (50 µl per well) and incubated at 37 °C in 5% CO_2_ for 20–24 h. In a separate plate, a dilution series of each agonist was prepared in Stimulation Buffer (Cisbio). The culture medium was removed and cells were incubated with 14 µl of agonist-containing Stimulation Buffer at 37 °C. After 1–2 h, 6 µl of Lysis and Detection Buffer (Cisbio) containing IP1-d2 and IP1 Tb cryptate antibody conjugates were added, and the reaction was incubated at room temperature protected from light. After 1 h, fluorescence was measured on an EnVision fluorescence plate reader (PerkinElmer) with the excitation wavelength set to 320 nm, and emission monitored at 620 nm (donor) and 665 nm (acceptor). Data were analyzed using GraphPad Prism.

### Molecular dynamics simulations

A model of OX_2_R bound to full-length OxB was built in Coot^63^ based on the cryo-EM structure of the OX_2_R–G-protein complex bound to the carboxy-terminal portion of OxB, using the weak density in the extracellular region as a guide. This model was comprised of residues F39–W251 and R292–A378. The disordered amino-terminal region and ICL3 were excluded due to missing density and available templates, while a complete ECL2, as well as residues F39–L48 of the amino-terminal α-helix of OX_2_R were included. This OX_2_R–OxB complex was prepared for MD simulations using Maestro (Schrödinger). Atomic coordinates from the model were imported and refined using the Protein Preparation Wizard with default parameters and settings. The protonation states were determined and set for all titratable residues at pH 7.4. Hydrogens were added to the system and then minimized. The OX_2_R–OxB complex model was aligned to the Orientation of Proteins in Membranes database structure of PDB 5WQC (http://opm.phar.umich.edu)^67^, inserted into a POPC bilayer and solvated using the System Builder Wizard in Maestro. Finally, water molecules (using the TIP3P water model) and ions were added, using the default parameters provided in the Maestro interface. MD simulations were performed using Desmond^68^ as included in the Schrödinger 2020-1 release, using OPLS3e force field for all atoms. Simulations were carried out in the NPT ensemble at 300 K and 1 bar. The default Desmond protocol was followed for relaxation. Restraints of 5 kcal mol^−1^ Å^−2^ were applied to the heavy atoms of those residues that are in direct contact with residues of the G protein at intracellular interface with OX_2_R to ensure that the receptor remained in an active conformation for the duration of the simulation. Individual simulations were run to a final production length of 1000 ns, with 2000 frames recorded from each independent replicate run for analysis. A total of four independent classical MD trajectories were run for an aggregate of 4 µs of simulation time.

### Reporting summary

Further information on research design is available in the Nature Research Reporting Summary linked to this article.

## Supplementary information

Supplementary Information

Supplementary Data 1

Reporting Summary

Description of Additional Supplementary Files

## Data Availability

The cryo-EM density maps for the OxB-bound and the compound **1**-bound OX_2_R–G-protein complexes have been deposited in the Electron Microscopy Data Bank under accession codes EMD-23118 and EMD-23119, and their coordinates are available from the Protein Data Bank under accession numbers 7L1U and 7L1V, respectively. All other data relating to this study are available from the corresponding authors upon reasonable request. Source data are provided with this paper.

## Source data

Source Data
