# Supplementary material for: Structures of active-state orexin receptor 2 rationalize peptide and small-molecule agonist recognition and receptor activation
Source: Nat Commun. 2021 Feb 5;12:815. doi: 10.1038/s41467-021-21087-6 (PMC7864924; doi:10.1038/s41467-021-21087-6)
Supplement: Supplementary file 1 — Supplementary Information [file 41467_2021_21087_MOESM1_ESM.pdf]

Supplementary Information

Structures of active-state orexin receptor 2 rationalize peptide and small-molecule agonist recognition and receptor activation

Chuan Hong[1,8], Noel J. Byrne[2,8], Beata Zamlynny[3], Srivanya Tummala[2], Li Xiao[1], Jennifer M. Shipman[2], Andrea T. Partridge[2], Christina Minnick[4], Michael J. Breslin[5], Michael T. Rudd[5], Shawn J. Stachel[5], Vanessa L. Rada[5], Jeffrey C. Kern[5], Kira A. Armacost[2], Scott A. Hollingsworth[6], Julie A. O'Brien[4], Dawn L. Hall[2], Terrence P. McDonald[7], Corey Strickland[1], Alexei Brooun[2], Stephen M. Soisson[2]* & Kaspar Hollenstein[2]*

[1]Computational & Structural Chemistry, MRL, Merck & Co., Inc., Kenilworth, NJ, USA.

[2]Computational & Structural Chemistry, MRL, Merck & Co., Inc., West Point, PA, USA.

[3]Screening & Compound Profiling, MRL, Merck & Co., Inc., Kenilworth, NJ, USA.

[4]Quantitative Bioscience, MRL, Merck & Co., Inc., West Point, PA, USA.

[5]Discovery Chemistry, MRL, Merck & Co., Inc., West Point, PA, USA.

[6]Computational & Structural Chemistry, MRL, Merck & Co., Inc., South San Francisco, CA, USA.

[7]Neuroscience, MRL, Merck & Co., Inc., West Point, PA, USA.

[8]These authors contributed equally: Chuan Hong, Noel J. Byrne.

*Two whom correspondence should be addressed:

stephen_soisson@merck.com (S.M.S.); kaspar.hollenstein@merck.com (K.H.)

Supplementary Tables 1 and 2

Supplementary Figures 1-11

Supplementary Table 1 Cryo-EM data collection, refinement and validation statistics.

	OxB (EMD-23118) (PDB 7L1U)	Compound **1** (EMD-23119) (PDB 7L1V)
**Data collection and processing**		
Magnification	59,524	59,524
Voltage (kV)	300	300
Electron exposure (e–/Å^2)	42.5	42.5
Defocus range (μm)	-0.6 to -2.2	-0.6 to -2.2
Pixel size (Å)	0.84	0.84
Symmetry imposed	C1	C1
Initial particle images (no.)	14,044,000	7,477,000
Final particle images (no.)	803,000	1,130,000
Map resolution (Å)	3.2	3.0
FSC threshold	0.143	0.143
Map resolution range (Å)	2.75-6.75	2.5-6.1
FSC threshold	0.5	0.5
**Refinement**		
Initial models used (PDB codes)	Refined structure with compound **1**	4S0V, 5G53, 3SN6, 6DDE, 3K1K
Model resolution (Å)	3.3	3.1
FSC threshold	0.5	0.5
Model resolution range (Å)	n/a	n/a
Map sharpening *B* factor (Å^2)	-131.2	-101.7
Model composition		
Non-hydrogen atoms	8,723	9,606
Protein residues	1,148	1,253
Ligands	0	1
B factors (Å^2)		
Protein	78.2	56.5
Ligand		52.1
R.m.s. deviations		
Bond lengths (Å)	0.011	0.007
Bond angles (°)	0.992	0.901
Validation		
MolProbity score	1.33	1.26
Clashscore	3.26	3.23
Poor rotamers (%)	0.78	0.30
Ramachandran plot		
Favored (%)	96.37	97.25
Allowed (%)	3.63	2.75
Disallowed (%)	0.00	0.00

Supplementary Table 2 Receptor-ligand interactions.

OxB	OX_2	Interaction
N20	$F333^{ECL3}$<br>$A334^{ECL3}$	sidechain-backbone hydrogen bond
H21	$R339^{7.28}$	backbone-sidechain hydrogen bond
A22	$K327^{6.58}$	backbone-sidechain hydrogen bond
	$F333^{ECL3}$	hydrophobic interactions
A23	$V209^{ECL2}$	hydrophobic interactions
G24	$D211^{ECL2}$	backbone-sidechain hydrogen bond
	$V114^{2.64}$<br>$V209^{ECL2}$	hydrophobic interactions
I25	$K327^{6.58}$	backbone-sidechain hydrogen bond
	$Y343^{7.32}$<br>$F346^{7.35}$	hydrophobic interactions
L26	$H350^{7.39}$	backbone-sidechain hydrogen bond
	$P131^{3.29}$<br>$Q134^{3.32}$<br>$C210^{ECL2}$	hydrophobic interactions
T27	$Q134^{3.32}$	backbone-sidechain hydrogen bond
	$N324^{6.55}$	sidechain-sidechain hydrogen bond
M28	$F227^{5.42}$<br>$T231^{5.46}$<br>$I320^{6.51}$<br>$H350^{7.39}$	hydrophobic interactions
	$Y354^{7.43}$	backbone-sidechain hydrogen bond

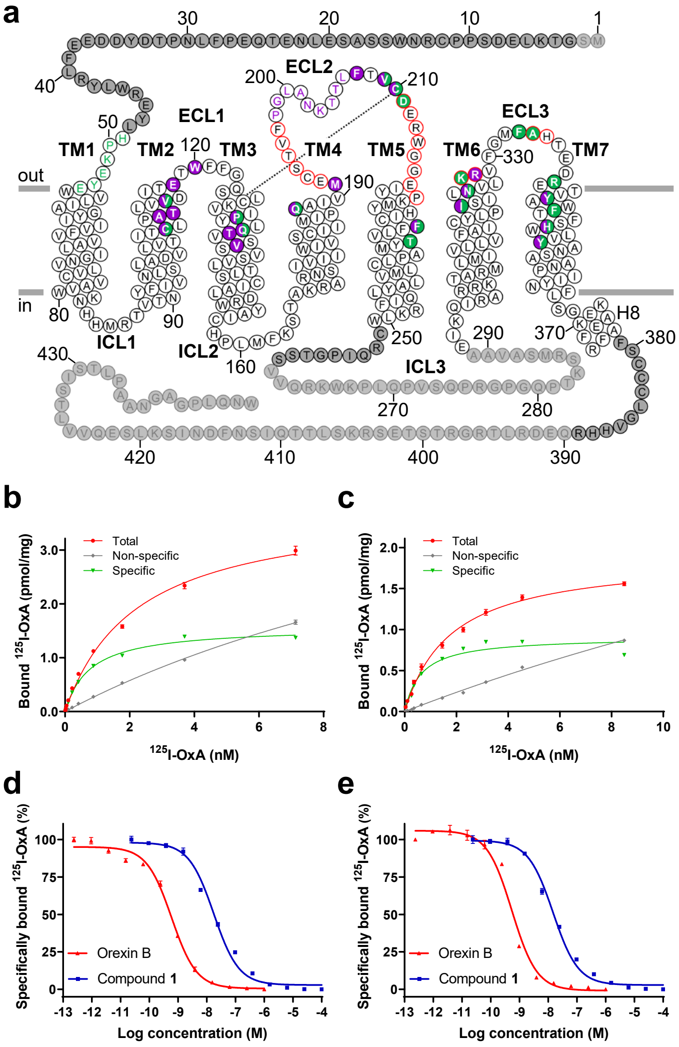

Supplementary Fig. 1 OX_2R construct used for structure determination. a, Schematic representation of human OX_2R. Residues not included in the engineered construct are shaded in light grey. Residues with green and purple letters are only included in the models with OxB and compound **1**, respectively, while those excluded from both models are shaded in dark grey. White letters on green and purple backgrounds denote residues in contact with OxB and compound **1**, respectively, while white letters on dual-color background interact with both agonists. Red circles indicate residues that form the Sb51 epitope. **b**, **c** Saturation binding of ^{125}I-OxA to wildtype and engineered OX_2R, respectively. Dissociation constants to wildtype and engineered OX_2R represented as pK_D are 9.12 ± 0.05 and 9.26 ± 0.17, respectively. **d**, **e** Competition binding studies with wildtype and engineered OX_2R, respectively, measuring the displacement of ^{125}I-OxA with increasing concentrations of compound **1** and OxB. Inhibition constants, represented as pK_i, to wildtype OX_2R are 9.25 ± 0.04 (OxB) and 7.78 ± 0.03 (compound **1**) and 9.29 ± 0.05 (OxB) and 7.86 ± 0.03 (compound **1**) to engineered OX_2R. Error bars represent the standard error of the mean of n = 3 independent experiments. Data are presented as mean values ± s.e.m. Source data are provided as a Source Data file.

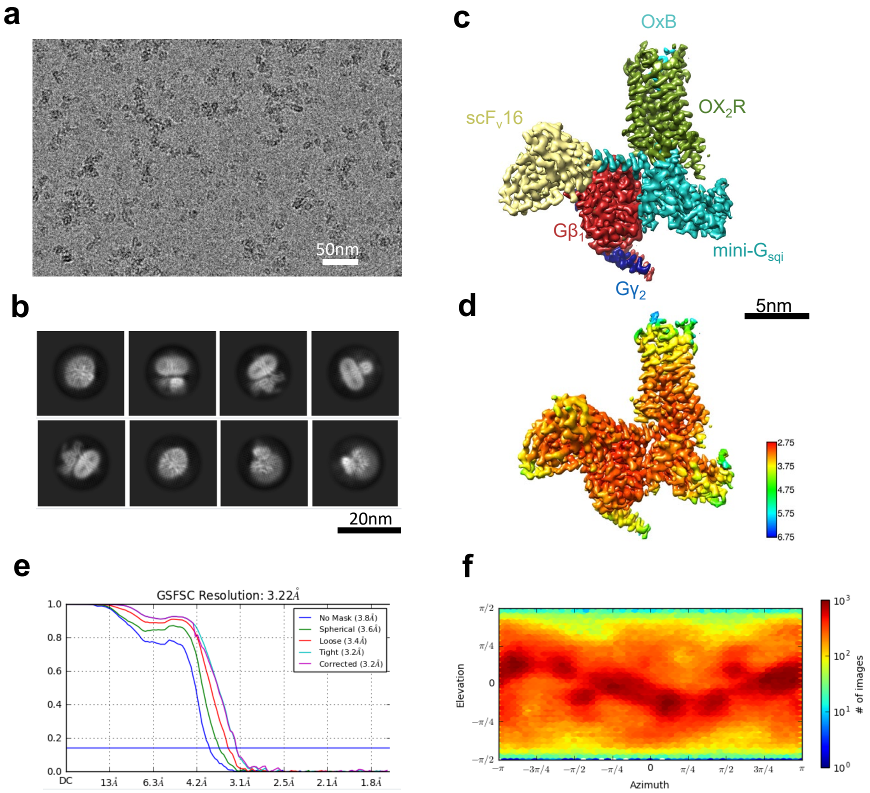

Supplementary Fig. 2 Cryo-EM data of OX_2R-G protein complex bound to OxB. a, b, Representative raw electron micrograph of a total of 38,810 movie stacks and 2D class averages, respectively. **c, d,** Electron density map of the OX_2R-G protein complex bound to OxB colored by subunit and local resolution estimate (2.75Å-6.75Å, 0.5 FSC cutoff), respectively. **e,** Gold standard FSC curves with correction of masking effects at 0.143 cutoff. **f,** Angular distribution heat map showing coverage of all orientations from the data.

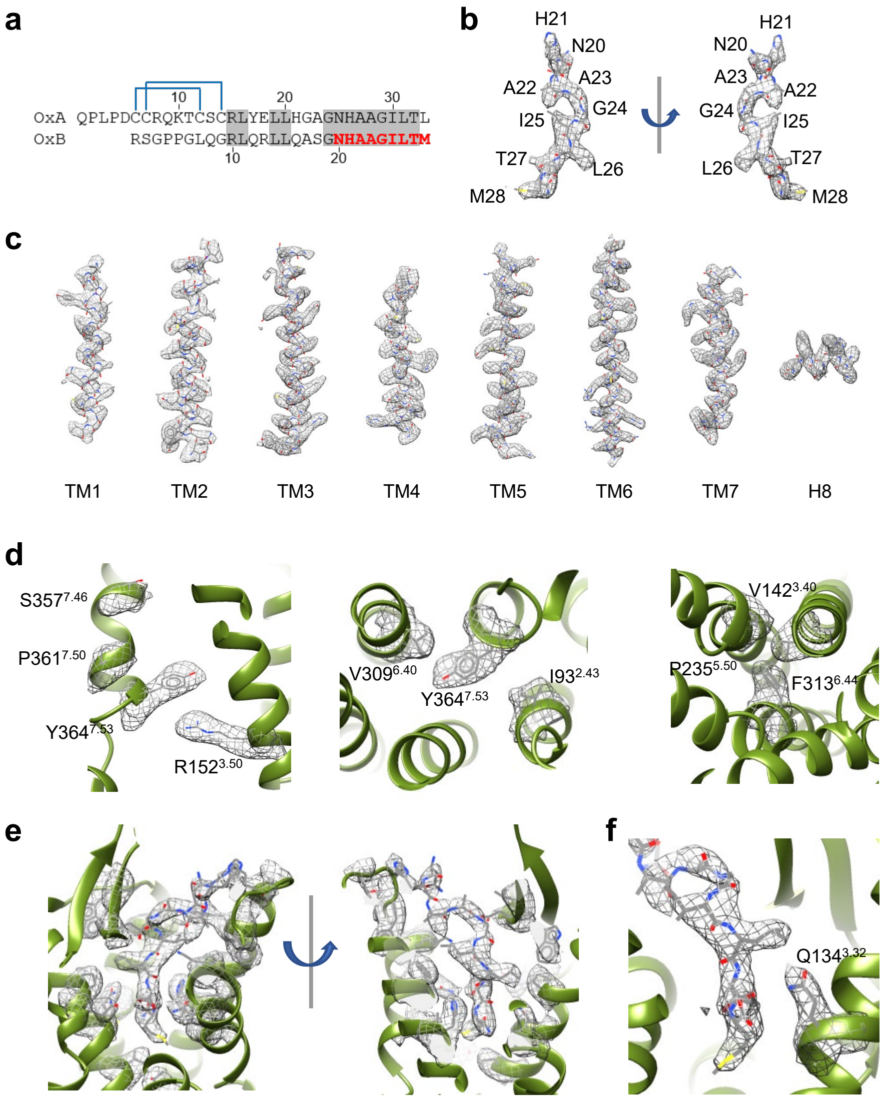

Supplementary Fig. 3 Electron density representation of OX_2R-G protein complex bound to OxB. a, Aligned amino-acid sequences of endogenous orexin agonists. The portion of the OxB sequence that is well-resolved in the cryo-EM map is highlighted in red. Disulfide bonds in OxA are indicated by blue lines. Identical residues are shaded in grey. **b,** Electron density map around residues N20-M28 of OxB viewed from two angles. **c,** Density representation for the seven transmembrane helices and helix 8 of OX_2R. **d,** Density views for microswitches DRY (left), NPxxY (middle), and hydrophobic core triad (right). **e,** Density views for OxB with contacting residues of the binding pocket. **f,** Density view for Q134$^{3.32}$.

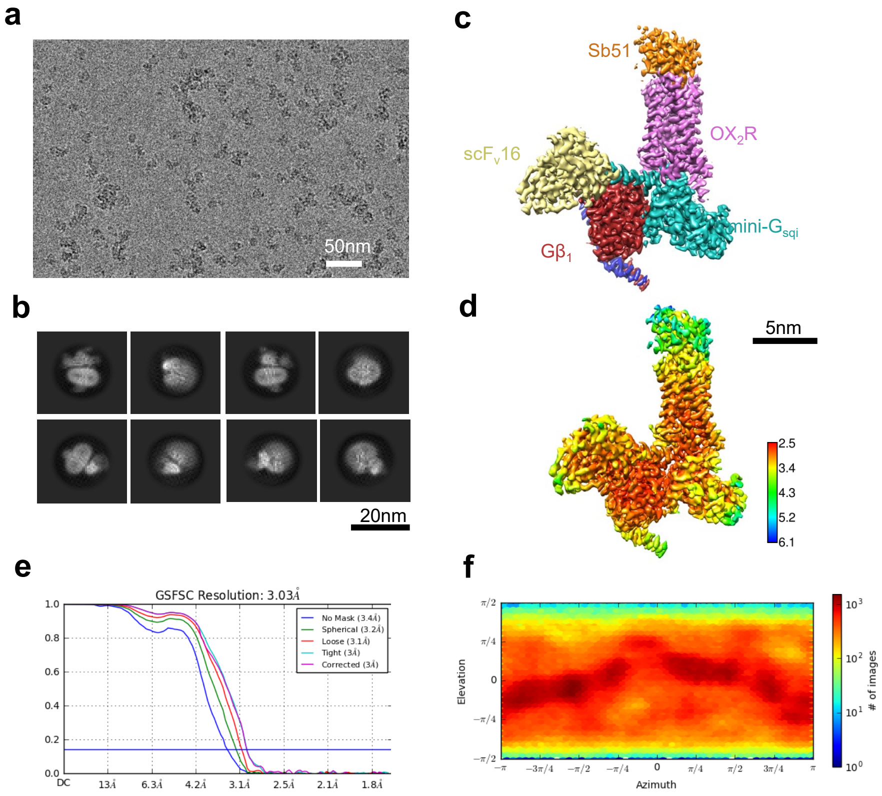

Supplementary Fig. 4 Cryo-EM data of OX_2R-G protein complex bound to compound 1. **a, b,** Representative raw electron micrograph of a total of 17,956 movie stacks and 2D class averages, respectively. **c, d,** Electron density map of the OX_2R-G protein complex bound to compound **1** colored by subunit and local resolution estimate (2.5Å-6.1Å, 0.5 FSC cutoff), respectively. **e,** Gold standard FSC curves with correction of masking effects at 0.143 cutoff. **f,** Angular distribution heat map showing coverage of all orientations from the data.

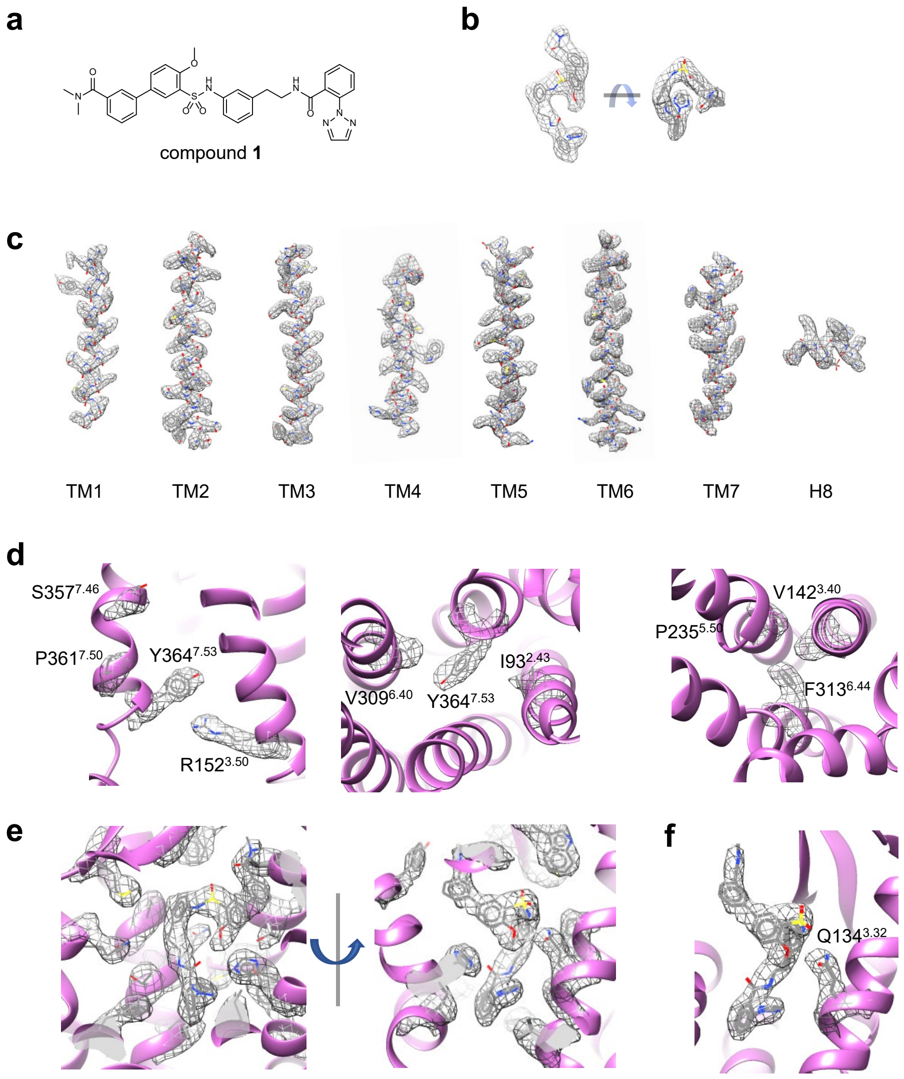

Supplementary Fig. 5 Electron density representation of OX_2R-G protein complex bound to compound 1. a, Structural formula of small-molecule orexin agonist used in this study. **b,** Electron density map around compound **1** viewed from two angles. **c,** Density representation for the seven transmembrane helices and helix 8 of OX_2R. **d,** Density views for microswitches DRY (left), NPxxY (middle), and hydrophobic core triad (right). **e**, Density views for compound **1** with contacting residues of the binding pocket. **f,** Density view for Q134$^{3.32}$.

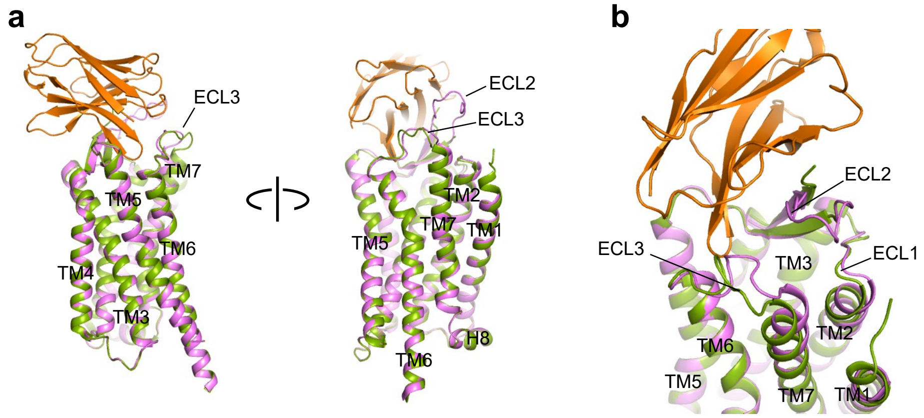

Supplementary Fig. 6 Structural differences between OxB- and compound 1-bound OX_2R. a, Superposition of the Cryo-EM structures of OX_2R bound to OxB (green) and compound **1** (purple) viewed from within the membrane from two different angles. Sb51 in complex with compound **1**-bound OX_2R is shown in orange. **b**, Close-up view from the extracellular side.

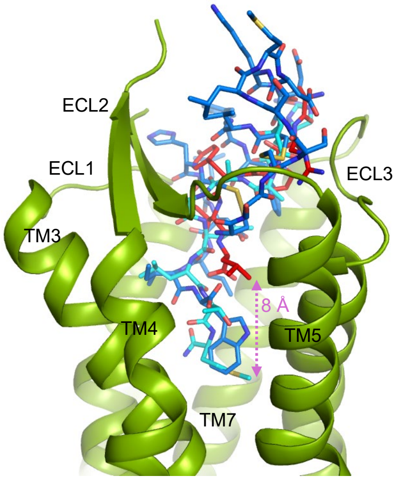

Supplementary Fig. 7 Comparison with endothelin 1 and neurotensin. Overlay of OX_2R (green) bound to OxB (cyan) with endothelin 1 (ET-1; blue) and neurotensin (NTS; red) after superposition of the 3 receptors (PDB IDs: 5GLH (ET_B receptor) and 4GRV (NTS_1 receptor). OxB and ET-1 penetrate approx. 8 Å deeper into the core of their respective receptors than NTS.

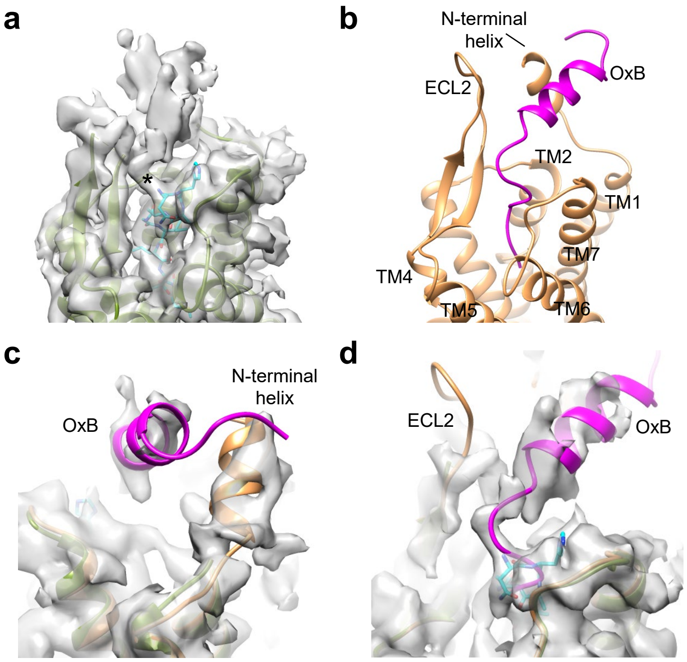

Supplementary Fig. 8 Receptor-peptide interactions at extracellular interface. a, Cryo-EM map (grey surface) at the extracellular surface of OX_2R (green). The position of the mainchain nitrogen of N20 of OxB (cyan) is indicated with an asterisk. **b,** Model of OX_2R (brown) in complex with full-length OxB (magenta). **c, d,** Close-up views of the cryo-EM density corresponding to the amino-terminal helix of OX_2R and the amino-terminal portion of OxB, respectively. Colors as in **a** and **b**.

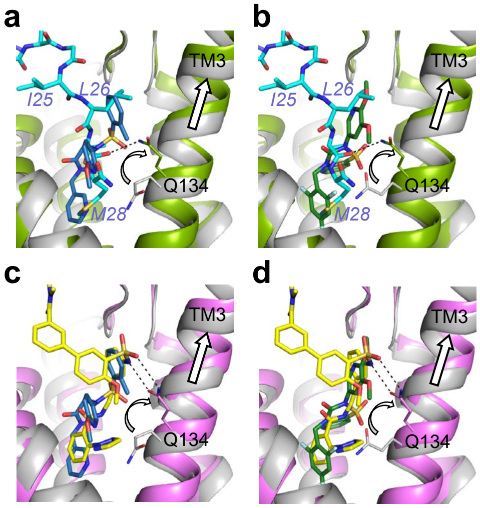

Supplementary Fig. 9 Comparison of agonist and antagonist binding. Superpositions of OX_2R in inactive (grey) and active (green and purple) conformations. **a, b,** Comparison of the binding mode of OxB (cyan) with those of the antagonists EMPA (blue; PDB ID 5WQC) and HTL6641 (dark green; 6TPN), respectively. **c, d,** Comparison of the binding mode of compound **1** (yellow) with those of EMPA and HTL6641, respectively. Conformational changes in TM3 and $Q134^{3.32}$ upon activation are highlighted by white arrows.

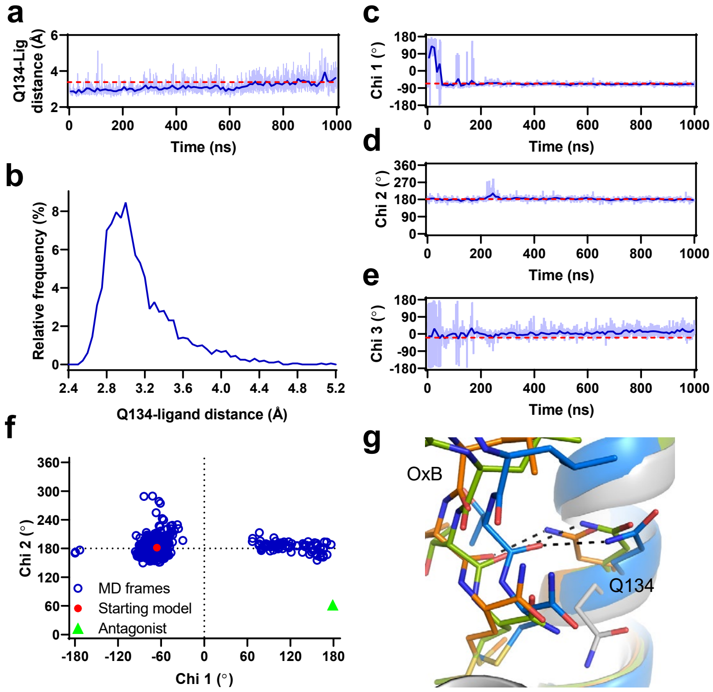

Supplementary Fig. 10 Conformational stability of the Q134$^{3.32}$ sidechain in molecular dynamics simulations. a, Distance between backbone carbonyl oxygen of T27 of OxB and the Q134$^{3.32}$ sidechain nitrogen of OX_2R over the course of the same 1000-ns MD simulation as shown in main Fig. 4. A central moving average (window length: 10 ns) is indicated by a dark blue line. **b,** Frequency distribution of the same distance as in **a**. **c-e,** Three sidechain torsion angles of Q134$^{3.32}$ plotted over the course of the same simulation as in main Fig. 4. Torsion angles are: N-Cα-Cβ-Cγ (Chi 1); Cα-Cβ-Cγ-Cδ (Chi 2), and Cβ-Cγ-Cδ-Nε (Chi 3). Note: As Chi 2 was found to hover near ±180 degrees, which results in a discontinuity when plotted as in **c,** Chi 2 was wrapped by adding 360 ° to all angles lower than 0 ° to maintain torsion angle continuity in the torsion evolution in **d** to better capture the stability observed in the simulation. The red dotted lines in **a** and **c-e** indicate the respective values of the starting model for reference. A central moving average (window length: 10 ns) is indicated by a dark blue line. **f,** 2D-plot of the Q134$^{3.32}$ torsion angles Chi 1 and Chi 2. Values of the latter were wrapped as in **d**. Angles of the starting model and of inactive-state OX_2R (PDB ID 5WQC) are indicated by a red circle and a green triangle, respectively. **g,** Overlay of the starting model of the microsecond MD simulations (green) with frames after 7 ns (orange) and 801 ns (blue) as well as the crystal structure of inactive-state OX_2R (gray; PDB ID 5WQC). Hydrogen bonds between the sidechain of Q134$^{3.32}$ to the mainchain carbonyl of OxB T27 are shown as black dotted lines.

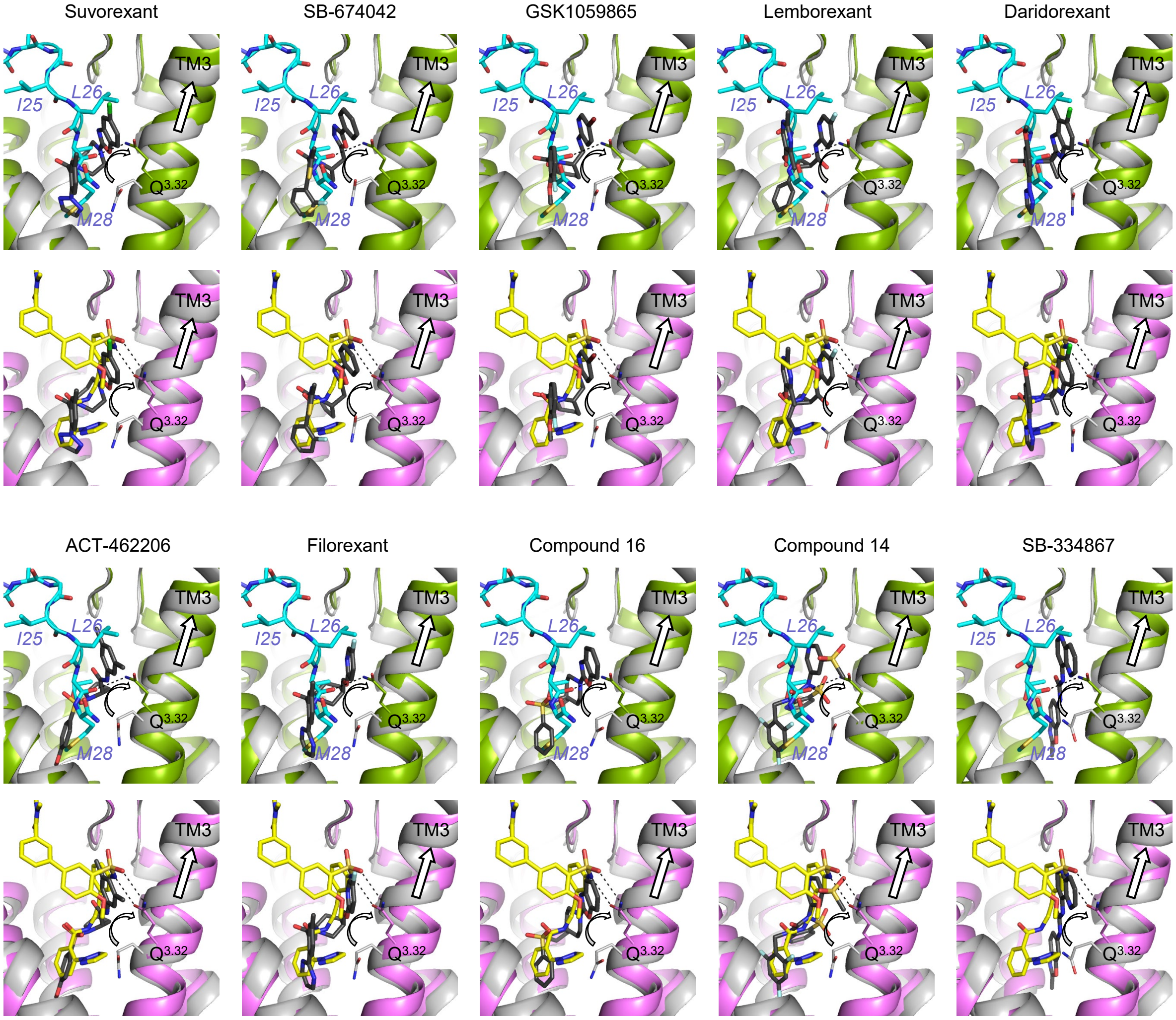

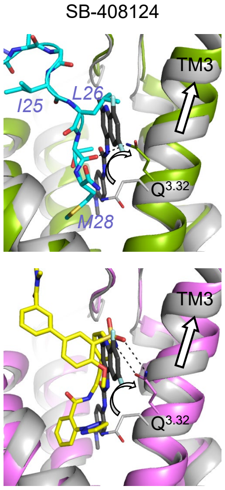

Supplementary Fig. 11 Comparison of agonist-bound OX_2R and antagonist-bound OX_1R. Superpositions of inactive-state OX_1R (grey) and active-state OX_2R (green and purple) are shown for 11 dual and single orexin antagonists. OxB, compound **1**, and antagonists are colored cyan, yellow, and dark grey, respectively. Conformational changes in TM3 and $Q^{3.32}$ upon activation are highlighted by white arrows. PDB IDs of antagonist-bound OX_1R structures are 4ZJ8 (suvorexant), 4ZJC (SB-674042), 6TOS (GSK1059865), 6TOT (lemborexant), 6TP3 (daridorexant), 6TP4 (ACT-462206), 6TP6 (filorexant), 6TQ4 (compound 16), 6TQ6 (compound 14), 6TQ7 (SB-334867), and 6TQ9 (SB-408124).
