# Supplementary material for: Structures of active-state orexin receptor 2 rationalize peptide and small-molecule agonist recognition and receptor activation
Source: Nat Commun. 2021 Feb 5;12:815. doi: 10.1038/s41467-021-21087-6 (PMC7864924; doi:10.1038/s41467-021-21087-6)
Supplement: Supplementary file 3 — Reporting Summary [file 41467_2021_21087_MOESM3_ESM.pdf]

nature research

Corresponding author(s): Kaspar Hollenstein
Stephen M. Soisson

Last updated by author(s): Dec 16, 2020

Reporting Summary

Nature Research wishes to improve the reproducibility of the work that we publish. This form provides structure for consistency and transparency in reporting. For further information on Nature Research policies, see our Editorial Policies and the Editorial Policy Checklist.

Statistics

For all statistical analyses, confirm that the following items are present in the figure legend, table legend, main text, or Methods section.

n/a	Confirmed	
☐	☒	The exact sample size (*n*) for each experimental group/condition, given as a discrete number and unit of measurement
☐	☒	A statement on whether measurements were taken from distinct samples or whether the same sample was measured repeatedly
☒	☐	The statistical test(s) used AND whether they are one- or two-sided<br>*Only common tests should be described solely by name; describe more complex techniques in the Methods section.*
☒	☐	A description of all covariates tested
☒	☐	A description of any assumptions or corrections, such as tests of normality and adjustment for multiple comparisons
☒	☐	A full description of the statistical parameters including central tendency (e.g. means) or other basic estimates (e.g. regression coefficient) AND variation (e.g. standard deviation) or associated estimates of uncertainty (e.g. confidence intervals)
☒	☐	For null hypothesis testing, the test statistic (e.g. *F*, *t*, *r*) with confidence intervals, effect sizes, degrees of freedom and *P* value noted<br>*Give P values as exact values whenever suitable.*
☒	☐	For Bayesian analysis, information on the choice of priors and Markov chain Monte Carlo settings
☒	☐	For hierarchical and complex designs, identification of the appropriate level for tests and full reporting of outcomes
☒	☐	Estimates of effect sizes (e.g. Cohen's *d*, Pearson's *r*), indicating how they were calculated

Our web collection on statistics for biologists contains articles on many of the points above.

Software and code

Policy information about availability of computer code

Data collection	Gatan Microscopy Suite Latitude v. 3.32.2403
Data analysis	CryoSPARC v. 2.15, Coot v. 0.8.9.2, Grade v. 1.2.19, Phenix v. 1.15, Molprobity (version implemented in Phenix v. 1.15), Schrödinger suite v. 2020-1, Desmod v. 6.1, PyMOL v. 2.1.1, UCSF Chimera v. 1.13.1, GraphPad Prism v. 8.1.1

For manuscripts utilizing custom algorithms or software that are central to the research but not yet described in published literature, software must be made available to editors and reviewers. We strongly encourage code deposition in a community repository (e.g. GitHub). See the Nature Research guidelines for submitting code & software for further information.

Data

Policy information about availability of data

All manuscripts must include a data availability statement. This statement should provide the following information, where applicable:

- Accession codes, unique identifiers, or web links for publicly available datasets
- A list of figures that have associated raw data
- A description of any restrictions on data availability

The cryo-EM density maps for the OxB-bound and the compound 1-bound OX2-G-protein complexes have been deposited in the Electron Microscopy Data Bank under accession codes EMD-23118 and EMD-23119 and their coordinates are available from the Protein Data Bank under accession numbers 7L1U and 7L1V, respectively. All other data relating to this study are available from the corresponding author upon reasonable request. Source Data are provided with this paper.

Field-specific reporting

Please select the one below that is the best fit for your research. If you are not sure, read the appropriate sections before making your selection.

☒ Life sciences ☐ Behavioural & social sciences ☐ Ecological, evolutionary & environmental sciences

For a reference copy of the document with all sections, see nature.com/documents/nr-reporting-summary-flat.pdf

Life sciences study design

All studies must disclose on these points even when the disclosure is negative.

Sample size	Sample size was not predetermined. For the cryo-EM structures, the number of images and particles used were determined by the amount of time available on the electron microscope and which gave us a high-resolution structure. Functional assays were repeated 4 times in parallel (n=4 independent experiments). Binding assays were repeated 3 times in parallel (n=3 independent experiments). This ensured an amount of data points which was statistically relevant (at least 3 repetitions per each experimental condition). These data were used to calculate mean and standard error of the mean.
Data exclusions	To obtain cryo-EM structures of high resolution, particle projections that did not align well with the major population were excluded form the data sets. No data were excluded in functional and binding assays.
Replication	Cryo-EM structure determination does not require replication as the structure represents an average structure calculated from a large number of molecules. Functional and binding assays had a number of independent experiments performed with an appropriate number of replicates (as reported in the manuscript). For functional and binding assays, all attempts at replication were successful.
Randomization	All variables could be controlled and so randomization was not required.
Blinding	Blinding is not possible in single particle cryo-EM as it is essential to have full information about the material in order to determine 3-dimensional structures. For functional and binding assays, the investigators were not blinded to the identity of the samples while performing the experiments because samples were generated, assayed, and analyzed by the same investigator.

Reporting for specific materials, systems and methods

We require information from authors about some types of materials, experimental systems and methods used in many studies. Here, indicate whether each material, system or method listed is relevant to your study. If you are not sure if a list item applies to your research, read the appropriate section before selecting a response.

Materials & experimental systems

n/a	Involved in the study	
☒	☐	Antibodies
☐	☒	Eukaryotic cell lines
☒	☐	Palaeontology and archaeology
☒	☐	Animals and other organisms
☒	☐	Human research participants
☒	☐	Clinical data
☒	☐	Dual use research of concern

Methods

n/a	Involved in the study	
☒	☐	ChIP-seq
☒	☐	Flow cytometry
☒	☐	MRI-based neuroimaging

Eukaryotic cell lines

Policy information about cell lines

Cell line source(s)	Expi293 cells were obtained from ThermoFisher. Trichoplusia ni insect cells were from Expression Systems. CHO cells stably expressing OX1R and OX2R, resp., were acquired from Aurora Biosciences.
Authentication	No authentication required
Mycoplasma contamination	All cell lines used in this study were negative for mycoplasma contamination.
Commonly misidentified lines (See ICLAC register)	No commonly misidentified cell lines were used.
