# Supplementary material for: Structures of active-state orexin receptor 2 rationalize peptide and small-molecule agonist recognition and receptor activation
Source: Nat Commun. 2021 Feb 5;12:815. doi: 10.1038/s41467-021-21087-6 (PMC7864924; doi:10.1038/s41467-021-21087-6)
Supplement: Supplementary file 4 — Description of Additional Supplementary Files [file 41467_2021_21087_MOESM4_ESM.docx]

Description of additional supplementary information

Title: Supplementary Data 1

Description: The file contains a table detailing the amino-acid sequences of the 57 synthetic nanobodies selected based on expression level and sample homogeneity after purification. The dissociation constants KD for the binding to agonist-bound OX2R are given if determined.
